# Combined Tuberculosis and Diabetes Mellitus Screening and Assessment of Glycaemic Control among Household Contacts of Tuberculosis Patients in Yangon, Myanmar

**DOI:** 10.3390/tropicalmed5030107

**Published:** 2020-06-29

**Authors:** Nyi-Nyi Zayar, Rassamee Sangthong, Saw Saw, Si Thu Aung, Virasakdi Chongsuvivatwong

**Affiliations:** 1 Epidemiology Unit, Faculty of Medicine, Prince of Songkla University, Hat Yai 90110, Thailand; nyinyi1987@gmail.com (N.-N.Z.); rassamee.s@psu.ac.th (R.S.); 2 Epidemiology Research Division, Department of Medical Research, Yangon 11191, Myanmar; 3 Department of Medical Research (Pyin Oo Lwin Branch), Pyin Oo Lwin 05085, Myanmar; sawsawsu@gmail.com; 4 Department of Public Health, Ministry of Health and Sports, Nay Pyi Taw 15015, Myanmar; sithuaung@mohs.gov.mm

**Keywords:** screening, tuberculosis, diabetes mellitus, contact investigation, TB-DM

## Abstract

Background: This study aimed to identify the prevalence of diabetes mellitus (DM) and tuberculosis (TB) among household contacts of index TB patients in Yangon, Myanmar. Method: Household contacts were approached at their home. Chest X-ray and capillary blood glucose tests were offered based on World Health Organization and American Diabetes Association guidelines. Crude prevalence and odds ratios of DM and TB among household contacts of TB patients with and without DM were calculated. Results: The overall prevalence of DM and TB among household contacts were (14.0%, 95% CI: 10.6–18.4) and (5%, 95% CI: 3.2–7.6), respectively. More than 25% of DM cases and almost 95% of TB cases among household contacts were newly diagnosed. Almost 64% of known DM cases among household contacts had poor glycaemic control. The risk of getting DM among household contacts of TB patients with DM was significantly higher (OR—2.13, 95% CI: 1.10–4.12) than those of TB patients without DM. There was no difference in prevalence of TB among household contacts of TB patients with and without DM. Conclusion: Significant proportions of the undetected and uncontrolled DM among household contacts of index TB patients indicate a strong need for DM screening and intervention in this TB–DM dual high-risk population.

## 1. Introduction

Globally, approximately 15% of tuberculosis (TB) patients are comorbid with diabetes mellitus (DM) [1]. Meanwhile, patients with DM have almost a three times higher risk of developing TB than the general population [2]. It is estimated that stopping the increasing prevalence of DM could prevent 6 million TB cases and 1.1 million TB deaths in 13 countries over 20 years [3]. Myanmar is ranked 10th among high TB burden countries, with an estimated annual TB incidence of 181,000 (95% CI: 119,000–256,000) [4]. The country is also ranked 9th among countries with the highest incidence of TB with DM comorbidity [5]. The TB incidence in the Yangon Region was 506/100,000 of the population, which was significantly higher than the global average 132/100,000 [6,7]. The prevalence of DM in the Yangon urban area was 12.1% [8], which was also higher than the global and regional averages [9]. It was found that the adjusted hazard ratio of TB was 2.09 among patients with DM over three years [10]. Therefore, the burden of TB is predicted to increase in the future, with an increased prevalence of DM in the Yangon Region.

It is known that the risks of getting TB and DM are higher among household contacts and family members. The prevalence of TB among household contacts was 4.5% in low and middle income countries [11]. Additionally, close blood relatives and spouses of patients with DM have more than a two times higher risk of developing DM due to sharing genetic susceptibility and/or sharing lifestyles, including physical inactivity, dietary habits associated with BMI, and central obesity [12,13,14]. Previous studies reported that DM is more prevalent among patients with TB compared to the general population [15]. Therefore, the prevalence of both TB and DM might be increased among household contacts of TB patients because of the high prevalence of DM among patients with TB. 

In Myanmar, household contact investigation of TB, implemented since 2011, is an effective screening method for undiagnosed TB among household contacts of patients with TB [16]. Screening of DM was launched in 2018, according to a WHO recommendation, among patients with TB aged 40 years and above in 32 townships in 23 districts of Myanmar [17]. However, there is no guidance on screening of DM among household contacts of index TB patients. Without appropriate intervention, the burden of future TB might be higher among household contacts with DM living in the same household with patients with TB in Yangon. In addition, the risk of TB is especially higher among patients with poorly controlled DM who are exposed to patients with active TB [18]. Therefore, integration of DM screening and glycaemic monitoring of DM in household contact investigations could uncover not only hidden TB patients but also the undiagnosed DM and poor glycaemic control DM patients who are at high risk of developing TB. Comparing the TB and DM prevalence among household contacts of TB index cases with and without DM allows us to examine whether the two groups had different risks and different levels of needs in screening. This study intended to examine the prevalence of DM and of TB among household contacts of patients with TB and compare this prevalence based on the DM status of index TB patients. The proportion of poor glycaemic control of known cases of DM among these contacts was also assessed.

## 2. Materials and Methods 

### 2.1. Study Design and Setting

A cross-sectional study was conducted in the Insein and North Okkalapa townships in the Yangon urban area from March to December 2018. These areas were selected for two reasons. First, they had the highest TB case notification rates in the Yangon region, with 387 and 248 per 100,000 population, respectively. Second, Yangon Region had the highest DM prevalence (12.1%) in Myanmar [8]. 

More than 80% of patients with TB living in the study townships are registered in the Township TB clinics and take regular treatment, while others are registered in clinics supported by local and international non-government organizations [19]. Existing routine household contact investigation for TB in these areas is carried out by basic health staff at the township level. The screening of signs and symptoms of TB followed by sputum smear and chest radiography (CXR) to confirm the diagnosis and a gene Xpert test to detect multidrug resistant TB (MDR-TB) were done in Township TB clinics. The findings from this study may give a clue to the situation on a larger scale throughout the country.

### 2.2. Study Sample 

The study included newly diagnosed sputum smear positive index TB patients, aged ≥ 25 years, registered in township TB clinics. The study recruited only patients with newly diagnosed TB who had never received TB treatment to avoid hyperglycaemia due to the TB treatment [20]. Pregnant women, HIV positive TB patients, MDR-TB patients and those who had no family members were excluded. The exclusion criteria were based on some medical conditions that may affect blood sugar or risk of having DM. Pregnant women may have gestational DM [21]. Consistently, people living with HIV and receiving treatment for HIV may have increased risk of type 2 DM [22]. Furthermore, patients with DM have lower risk of developing MDR-TB compared to drug sensitive TB [2,23]. Therefore, the study excluded them to maintain homogeneity among the index cases. Family members living in the same households with an index TB patient for at least 3 months before having diagnosis of TB were invited for a contact investigation of TB. Among them, household contacts aged < 25 years were excluded for DM screening because the prevalence of DM increases steadily from age 25 years [24]. 

### 2.3. Sample Size Calculation

The sample size was calculated to test that the prevalence of TB among household contacts with DM (28%) was higher than that without DM (13%) [25]. At least 259 household contacts of index TB patients without DM and 65 household contacts of index TB patients with DM were required.

### 2.4. Data Collection Tools

A set of structured questionnaires was modified from previous research on household contact investigation in Myanmar [25] and a manual for WHO STEPwise approach to surveillance of non-communicable diseases and their risk factors [26]. 

The questionnaire included (1) Sociodemographic characteristics, such as age, gender, education, employment status and daily income per household member. Daily income per household member was converted from Myanmar kyats into United States dollars (USD) and cut off values for income level were 1.9 and 3.1 USD/day based on the poverty level defined by the World Bank [27]. (2) Signs and symptoms of TB, including evening rise in temperature, cough, coughing up blood, breathlessness, chest pain and weight loss. (3) Previous history of TB and DM (history of TB and/or DM diagnosed by a medical doctor). (4) Risk factors of TB (closeness with index TB patients including sharing the same room, sleeping in the same bed and taking care of index TB patients) and risk factors of DM (family history of DM, low physical activity: defined as <3 days of vigorous-level activity (e.g., carrying heavy loads, running, etc.,) of at least 20 min/week, or <5 days of moderate-level activity (e.g., walking very briskly, performing domestic chores, etc.,) using standard metabolic equivalents [28]. 

A physical examination including body mass index (BMI) was calculated using weight and height (kg/m^2^) and classified < 18.5 kg/m^2^ as underweight, 18.5–24.9 as normal, 25.0–29.9 kg/m^2^ as overweight and ≥30 kg/m^2^ as obese [29], and central obesity defined for those with waist circumference ≥ 94 cm in men and ≥80 cm in women [30], were also done during household visits.

### 2.5. Diagnosis of DM in Index TB Patients and Household Contacts

Both index TB patients and their household contacts were asked about their previous history of DM diagnosed by a medical doctor. The diagnosis was used as a confirmation of DM. They were also tested for their glycaemic control by fasting capillary blood glucose (FBG). Those without history of DM were tested by random capillary blood glucose (RBG) followed by FBG on the following day for TB index patients, and within 2 weeks for household contacts at township TB clinics. In case a contact did not revisit the clinics within 2 weeks, researchers then visited them at their home within 4 weeks to perform an FBG test. Any single positive test result was repeated on a separate day. All blood tests were done by an Accu-Chek^®^ Performa glucometer [31].

Based on the American Diabetes Association [32], DM was defined as subjects with known DM or newly diagnosed DM with RBG ≥ 200 mg/dl and FBG ≥ 126 mg/dl (or) RBG ≥ 200 mg/dl for two times on separate days (or) FBG ≥ 126 mg/dl for two times on separate days. Poor glycaemic control was defined as FBG ≥ 130 mg/dl among known cases of DM [33].

All participants were duly informed of their results. New patients with DM were referred to DM clinics for further management. 

### 2.6. Diagnosis of TB among Household Contacts

Household contacts were firstly screened by (1) asking about signs and symptoms of TB and (2) taking CXR at township TB clinics. 

For household contacts with at least one signs and symptoms of TB (or) abnormal CXR suggested of TB, smear microscopy test was done by collecting one spot sputum specimen at the time of visit to TB clinic and next-day early morning sputum specimen. Gene Xpert test was done for those who had at least one positive sputum smear microscopy result. 

For those who could not produce sputum, a 2-week trial of broad-spectrum antibiotics was prescribed. Disappearance of abnormalities on CXR would indicate non-TB. Otherwise, the subject was classified as TB. All contacts who were deemed to have positive TB were invited to register in the township TB clinics, where treatment would be provided according to the National TB treatment guideline.

Confirmation of TB could be made by 2 criteria. First, bacteriological confirmation using either a sputum smear or GeneXpert tests [34]. Second, a clinical diagnosis for those who did not meet the first criteria but had abnormal CXR. All contacts with newly diagnosed TB were asked to receive a full course of treatment [34].

### 2.7. Data Collection Procedure

Eligible patients registered in each township’s TB clinic were invited to participate in the study. After informed consent, a face-to-face interview and a blood investigation for DM were done. Participants were then asked for their permission to visit their home and screen their household contacts for TB and DM. 

### 2.8. Data Analysis

Epidata version 3.1 was used for data entry and R software was used for data analysis. The prevalence of TB or DM among household contacts was calculated by dividing the number of contacts with TB or DM by the number of household contacts screened for TB or DM. Chi-square test and Fisher’s exact test were used to test differences between the prevalence of TB or DM among household contacts of TB patients with and without DM. 

Multivariate logistic regression models were done to determine the odds ratios of getting DM and TB among household contacts based on the DM status of index TB patients, adjusted for covariates identified in previous studies [14,35,36,37]. Covariates included age, gender, socioeconomic status (SES), low physical activity, BMI, central obesity for risk of DM, age, gender, SES and closeness to index TB patient for risk of TB. These covariates were added cumulatively into each model. The model with the lowest Akaike’s information criterion (AIC) value indicated the best fitting model. All *p* values < 0.05 were taken as statistically significant. 

### 2.9. Ethical Approval

Ethical approval was obtained by the Ethics Review Committee at Prince of Songkla University (33/2017) and the Ethics Review Committee at the Department of Medical Research, Myanmar (023/2018).

## 3. Results

### 3.1. Investigation for DM among Index TB Patients

#### Prevalence of DM among Index TB Patients

**Figure 1:**
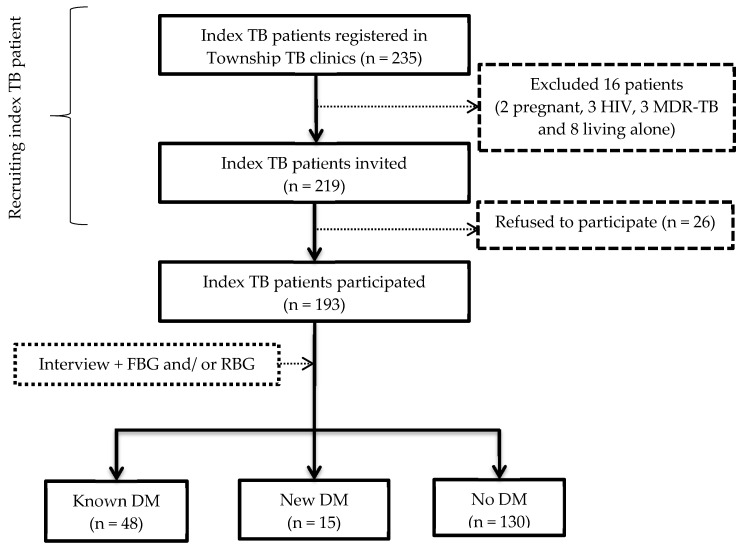
Flow chart of recruiting and investigation for diabetes mellitus (DM) among newly diagnosed index tuberculosis (TB) patients. MDR-TB—Multidrug resistant TB; RBG—Random capillary blood glucose; FBG—Fasting capillary blood glucose; Known DM—Patients with previous history of DM diagnosed by a medical doctor; New DM—Newly diagnosed DM patients (RBG ≥ 200 mg/dl & FBG ≥ 126 mg/dl (or) RBG ≥ 200 mg/dl for two times on separate days (or) FBG ≥ 126 mg/dl for two times on separate days).

Figure 1 shows a flow chart of the study. A total of 235 index TB patients were registered in the township TB clinics during the study period. Among them, 16 were excluded for various reasons. A total of 219 patients were invited and 193 agreed to participate (88% response rate). Among the 193 index TB patients, the prevalence with 95% confidence interval (CI) of known DM, newly diagnosed DM and overall DM were 24.9% (18.9–31.5), 7.8% (4.4–12.5) and 32.6% (26.1–39.7), respectively. Eight (12.7%) patients were aged between 29 and 39 years.

### 3.2. Prevalence and Glycaemic Control of DM among Household Contacts

**Figure 2:**
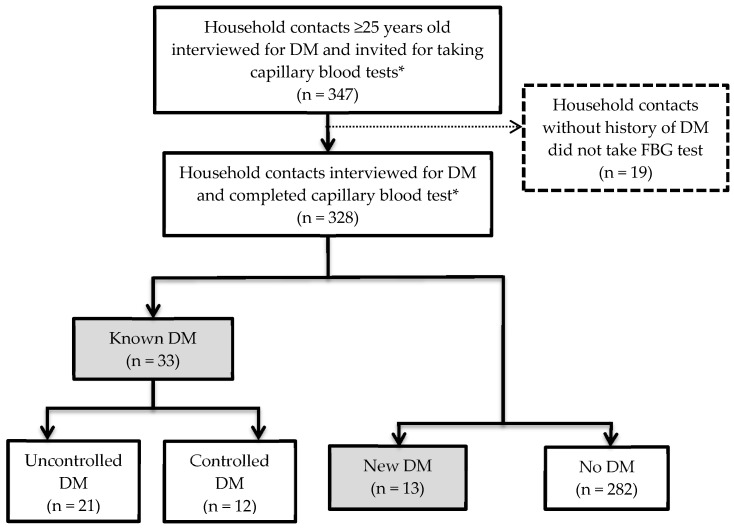
Flow chart for investigation for diabetes mellitus (DM) among household contacts. * Capillary blood test—Only fasting capillary blood glucose (FBG) test was done for known DM; and both random capillary blood glucose (RBG) test and FBG test were done for contacts without history of DM; Known DM—Household contacts with previous history of DM diagnosed by a health care personnel; Uncontrolled DM—FBG ≥ 130 mg/dl among known DM; New DM—Newly diagnosed DM (RBG ≥ 200 mg/dl & FBG ≥ 126 mg/dl (or) RBG ≥ 200 mg/dl for two times on separate days (or) FBG ≥ 126 mg/dl for two times on separate days).

Figure 2 shows a flow chart of the 347 household contacts aged 25 years and above who were investigated for DM. Among them, 33 had history of diagnosed with DM. Among the 314 household contacts without a history of DM and invited to have an FBG test in township TB clinics, 139 (44.3%) attended. The remaining 156 contacts (49.7%) had their FBG tested at their home during the household revisit.

In total, 328 contacts completed all DM investigations. Among these, 33 (10.1%) were known to have DM and 13 (4.0%) were newly diagnosed, resulting in an overall prevalence of DM among household contacts of 14.0%. Most (94%) of the contacts with DM were aged 40 years and above. Among the 33 known cases, 21 (63.6%) had poor glycaemic control.

### 3.3. Prevalence of TB among Household Contacts

**Figure 3:**
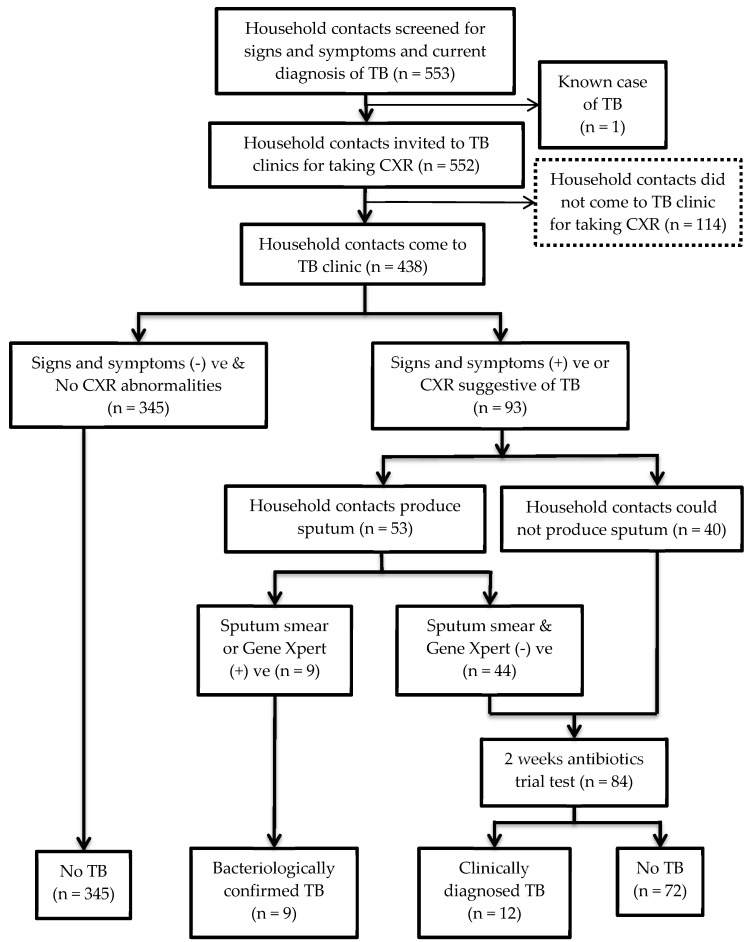
Flow chart for investigation for tuberculosis (TB) among household contacts.

Figure 3 shows a flow chart of the 553 household contacts who were investigated for TB. One household contacts among them was known case of TB. All household contacts, except the known case of TB, were invited to township TB clinics to have CXR regardless of having signs and symptoms of TB. Among them, 249 (45.1%) complied after the 1st home visit and 190 (34.4%) complied after the 2nd visit.

Overall, 439 (79.4%) completed all TB investigation. One (0.2%) was a known case and 21 (4.8%) were newly diagnosed, resulting in an overall TB prevalence of 5.0% among household contacts. MDR-TB was not detected.

### 3.4. Comparing Prevalence of DM and TB among Household Contacts of Index TB Patients with and without DM

Table 1 compares the prevalence of DM and TB among household contacts of TB patients with and without DM. The prevalence of DM, including known and newly diagnosed cases, was higher among household contacts of index TB with DM, although the difference was statistically significant only for the overall prevalence of DM. The prevalence of TB among household contacts, however, was comparable between the index TB with DM and without DM groups.

### 3.5. Number of Patients with DM among Household Contacts ≥ 25 Years Old

Among household contacts aged ≥ 25 years without TB, 32 (12.1%) were diagnosed with DM, including all newly diagnosed cases.

Among household contacts aged ≥ 25 years with TB, one case was found to have existing DM with poor glycaemic control.

### 3.6. Odds Ratio of Getting DM and TB among Household Contacts Based on DM Status of Index TB Patients

Table 2 shows the odds ratios of obtaining DM and TB among household contacts, based on the DM status of index TB patients adjusted for covariates. From models D1 to D7, household contacts of index TB patients with DM had significantly higher risks of getting DM than household contacts of index TB patients without DM. Among these models, D2 had the lowest AIC. Based on this model, household contacts of index TB–DM patients had 2.13 times higher risk of getting DM than household contacts of index TB patients without DM. 

From models T1 to T5, the risk of getting TB was lower in household contacts of index TB patients with DM, although the risk was not significantly different.

## 4. Discussion

In this study, comorbidity with DM among 32.6% of index TB patients highlighted the increased double burden of TB and DM in Yangon. The adherence to DM and TB screening among household contacts was increased with repeated home visits. Among household contacts, the prevalence of DM and TB were 14.0 and 5.0%, respectively, and more than a quarter of DM and 95% of TB were newly diagnosed. Among household contacts with existing DM, almost 63.6% had poor glycaemic control. The risk of DM among household contacts of index TB patients with DM was two times higher than those of TB patients without DM. There was no difference in the prevalence of TB among household contacts of both index TB patient groups. 

The adherence to having an FBG test in a TB clinic after the 1st home visit was 44.3% and an additional 49.7% were tested in their homes during the 2nd revisit. Therefore, testing FBG during household visits increased the screening coverage of household contacts. The adherence of household contacts to TB investigation in clinics after the 1st home visit was similar to a study conducted in South Africa (45.1%) [38]. The adherence rate in our study was increased up to 79.5% after the 2nd visit. Therefore, repeated home visits achieved more than 30% adherence to TB investigation.

Among index TB patients, the prevalence of DM, 32.6% (95% CI: 26.1–39.7) was significantly higher than that in the general population 12.1% (95% CI: 8.4–17.0) [8]. After adjustment for age, the risk of DM among index TB patients was 2.8 times greater than the risk of DM in the general population. This finding is similar to the results of a meta-analysis, where the risk of DM was found to be three times higher in TB patients [2]. In the current study, the prevalence of newly diagnosed DM among 29 to 39 year old TB patients was almost 13% of all newly diagnosed DM cases. The current Myanmar guideline on screening for DM confined to those aged ≥ 40 years for TB patients would miss nearly 13% of the DM cases among TB patients in the Yangon Region. 

Among household contacts who were aged 25 years or older, 14.0% (95% CI: 10.6–18.4) had DM, which was slightly higher than the 12.1% (95% CI: 8.4–17.0) prevalence in the general population [8]. After adjustment for age, no significant difference was found between the risk of DM among household contacts of TB patients and the risk of DM in the general population. Around 94% of patients with DM among household contacts were aged 40 years or over, which was similar with a previous Indian study, where household contacts aged 35 years and above comprised almost 93% of total DM patients [39]. Household contacts of TB–DM patients were more likely to have DM than those of TB patients without DM. This could be explained by genetic linkage similarity in risk behaviours among subjects in the same household. 

Our contact investigation could identify around 4.0% of new DM. In a survey in the Yangon urban area, 7.6% of the participants were identified as new DM based on a one time FBG test [8]. In our study, confirmation of DM was defined based on results of both RBG and FBG tests. Therefore, the specificity of the test is higher than that of either test [40]. The prevalence of DM among household contacts without TB was 12.1%, including all newly diagnosed DM patients. These newly diagnosed DM patients comprised almost 25% of all DM patients among household contacts. Although integration of DM screening in household contacts of TB patients increases the case detection rate of a relatively small percentage of hidden DM cases in a community, newly detected DM cases are more at risk to TB than those detected by other screening methods.

Our overall prevalence of TB among household contacts (5.0%) was similar with a systematic review of contact investigation done in low and middle-income countries [11]. The prevalence was higher than studies done in China (3.8%) estimated by the same method [41], but lower than the 13.8% found in a study done in Mandalay region, Myanmar [25]. The difference in prevalence of TB among household contacts can be explained by different TB backgrounds in different populations, and the lifestyles and living conditions of people in each area [42]. There was no significant difference in prevalence of TB among household contacts of index TB patients with and without DM. This result is similar to a previous one reported from India [39].

In the current study, nearly two-thirds of known DM cases among household contacts had poor glycaemic control. The corresponding percentage was 54% among the general population but the cut off value for poor glycaemic control was FBG ≥ 126 mg/dl, which was lower than FBG ≥ 130 mg/dl in our study [8]. A previous study conducted in the US found that uncontrolled DM patients continued to have a significantly elevated risk of TB infection (OR, 2.6; 95% CI, 1.5–4.6) compared to nondiabetics [43]. A study conducted in Taiwan reported that DM patients with poor glycaemic control had a significantly higher hazard of TB than those without DM during a median follow-up time of 4.6 years [18]. In the current study, screening of DM was done among household contacts of recently diagnosed TB patients and only one TB case was identified among household contacts with poorly controlled DM. Therefore, these DM patients need good follow up to improve their DM conditions and reduce the risk of developing TB in the future. 

## 5. Limitations

Our screening of DM was based on the finger prick method due to limited resources. The prevalence of DM may be different had the standard glucose concentrations in plasma been used [31,44]. Screening using CXR without sputum culture among those whose sputum could not be obtained might have led to an overestimated prevalence of TB among our contacts. 

## 6. Conclusions

Despite these limitations, our findings indicate that screening of DM during household visits increased feasibility and repeated household visits improved the adherence of TB screening using CXR among household contacts. In a high TB and DM co-prevalent area, DM screening and glycaemic control assessment for TB household contacts aged 40 years or over should be integrated with routine TB screening programmes.

## Figures and Tables

**Table 1:** Comparison of prevalence of overall and newly diagnosed DM and TB among household contacts of TB patients with and without DM.

**DM Screening in Household Contacts**	**Total**	**Household Contacts of Index TB with DM**	**Household Contacts of Index TB without DM**	***p* Value**
Number of household and household contact				
Total number of households visited (a)	193	63	130	N/A
Total number of household contacts screened for DM * (b)	328	104	224	N/A
Number of DM patients				
Known case of DM (c)	33	15	18	N/A
Newly diagnosed DM (d)	13	6	7	N/A
Prevalence of DM among household contacts, % (95%CI)				
Overall DM ((c + d)/b)	14.0 (10.6–18.4)	20.2 (13.2–29.4)	11.2 (7.5–16.2)	0.03
Known case of DM (c/b)	10.1 (7.1,13.9)	14.4 (8.6–22.9)	8.0 (4.9–12.6)	0.07
Newly diagnosed DM (d/b)	4.0 (2.2–6.9)	5.8 (2.4–12.6)	3.1 (1.4–6.6)	0.36 ^†^
**TB Screening in Household Contacts**	**Total**	**Household Contacts of Index TB with DM**	**Household Contacts of Index TB without DM**	***p* Value**
Number of household and household contact				
Total number of households visited (a)	193	63	130	N/A
Total number of household contacts screened for TB (b)	439	134	305	N/A
Number of TB patient				
Known case of TB (c)	1	0	1	N/A
Newly diagnosed TB (d)	21	6	15	N/A
Prevalence of TB among household contacts, % (95%CI)				
Overall TB prevalence ((c + d)/b)	5.0 (3.2–7.6)	4.5 (1.8–9.9)	5.3 (3.1–8.6)	0.73
Newly diagnosed TB (d/b)	4.8 (3.1–7.3)	4.5 (1.9–10.0)	4.9 (2.9–8.2)	0.84

N/A–Not applicable; * including both existing DM and household contacts who completed both RBG and FBG tests; CI—confidence interval; ^†^ Fisher’s exact test.

**Table 2:** Odds of getting DM and TB among household contacts based on the DM status of index TB patients in combination with various covariates.

**Outcome—DM Status of Household Contacts**									
**Model**	**Main Hypothesis Exposure**	**Covariate Included in the Model**						**OR (95% CI)**	**AIC**
	**DM in Index TB Patient**	**Age**	**Gender**	**SES**	**Low Physical Activity**	**BMI**	**Central Obesity**		
D1	+	-	-	-	-	-	-	2.01 (1.07–3.79) *	265.4
D2	+	+	-	-	-	-	-	2.13 (1.10–4.12) *	248.1
D3	+	+	+	-	-	-	-	2.24 (1.15–4.35) *	248.3
D4	+	+	+	+	-	-	-	2.39 (1.22–4.72) *	254.0
D5	+	+	+	+	+	-	-	2.37 (1.19–4.69) *	253.5
D6	+	+	+	+	+	+	-	2.27 (1.13–4.56) *	253.5
D7	+	+	+	+	+	+	+	2.28 (1.13–4.57) *	254.6
**Outcome—Active TB Status of Household Contacts**									
**Model**	**Main Hypothesis Exposure**	**Covariate Included in the Model**						**OR (95% CI)**	**AIC**
	**DM in Index TB Patient**	**Age**	**Gender**	**SES**	**Closeness to Index TB Patient**				
T1	+	-	-	-	-			0.85 (0.32–2.21)	178.5
T2	+	+	-	-	-			0.85 (0.33–2.23)	178.1
T3	+	+	+	-	-			0.82 (0.31–2.17)	175.3
T4	+	+	+	+	-			0.81 (0.30–2.21)	174.4
T5	+	+	+	+	+			0.87 (0.31–2.39)	175.7

OR—odds ratio; CI—confidence interval; AIC—Akaike’s information criterion; * *p* value < 0.05; SES—Socioeconomic status including formal education, employment and daily income per household member; Closeness with TB patients—sharing same room, sleeping in same bed and taking care of index TB patients.
